# Synthesis, crystal structure and Hirshfeld surface analysis of (3a*SR*,10*RS*,10a*RS*)-2-(4-iodo­phen­yl)-1-oxo-5-tosyl-1,2,3,3a,4,5,10,10a-octa­hydro­pyrrolo[3,4-*b*]carbazole-10-carb­oxy­lic acid–ethanol (4/1)

**DOI:** 10.1107/S2056989025011582

**Published:** 2026-01-06

**Authors:** Elizaveta D. Yakovleva, Atash V. Gurbanov, Victor N. Khrustalev, Mohammed Hadi Al-Douh, Tuncer Hökelek, Khudayar I. Hasanov, Roman A. Litvinov

**Affiliations:** a RUDN University, 6 Miklukho-Maklaya St., Moscow 117198, Russian Federation; b Excellence Center, Baku State University, Z. Khalilov Str. 33, AZ 1148, Baku, Azerbaijan; c Zelinsky Institute of Organic Chemistry of RAS, 47 Leninsky Prospect, 119991 Moscow, Russian Federation; d Chemistry Department, Faculty of Science, Hadhramout University, Mukalla, Hadhramout, Yemen; e Hacettepe University, Department of Physics, 06800 Beytepe-Ankara, Türkiye; f Azerbaijan Medical University, Scientific Research Centre (SRC), A. Kasumzade St. 14, AZ 1022, Baku, Azerbaijan; g Volgograd State Medical University, 1 Pavshikh Bortsov Sq., Volgograd 400131, Russian Federation; h LLC <<InnoVVita>>, Office 401, Room 2, 6 Komsomolskaya St., Volgograd 400066, Russian Federation; University of Buenos Aires, Argentina

**Keywords:** crystal structure, pyrrolo ring, carbazole, Hirshfeld surface analysis

## Abstract

The asymmetric unit of the title compound, 4C_28_H_23_IN_2_O_5_S·C_2_H_6_O, contains two crystallographically independent mol­ecules and an ethanol solvent mol­ecule. In the crystal, O—H⋯O and C—H⋯O hydrogen bonds link the mol­ecules into a three-dimensional architecture, enclosing *R*^4^_4_(23) ring motifs. C—H⋯π(ring) inter­actions and the π–π stacking between the parallel rings help to consolidate the packing. Hirshfeld surface analysis reveals that the most important contributions to the crystal packing are from H⋯H (36.1% and 38.5%), H⋯O/O⋯H (23.7% and 22.1%), H⋯C/C⋯H (20.0% and 16.1%) and H⋯I/I⋯H (6.4% and 10.1%) inter­actions.

## Abstract 2

The asymmetric unit of the title compound contains two crystallographically independent mol­ecules and an ethanol solvent mol­ecule. In the crystal, O—H⋯O and C—H⋯O hydrogen bonds link the mol­ecules into a three-dimensional architecture, enclosing *R*^4^_4_(23) ring motifs. C—H⋯π(ring) inter­actions and the π–π stacking between the centroids of the parallel rings help to consolidate the packing.

## 1. Chemical context

The development of novel compounds capable of preventing the disabling consequences of fibrotic remodeling diseases represents a highly promising direction in pharmacology (Lapthorn *et al.*, 2024 ▸). Oxidative stress is among the key factors contributing to the progression of such pathologies (Cheresh *et al.*, 2013 ▸). The annulated iso­indole scaffold may hold considerable potential, as evidenced by data on structurally related iso­indole motifs that have demonstrated anti­oxidant and anti­fibrotic effects under experimental conditions (Yakan *et al.*, 2023 ▸; Li *et al.*, 2013 ▸). Prior studies have provided evidence supporting the potential of hydrogenated iso­indole-7-carb­oxy­lic acids as a scaffold for the rational design of novel agents targeting diseases associated with non-enzymatic mol­ecular damage pathways (*e.g.*, glycation/glycoxidation) and oxidative stress, with the putative mode of action involving inhibition of oxidative processes (in particular, non-enzymatic glycation, some mechanistic steps of which are oxidation-dependent; Ibragimova *et al.*, 2024 ▸). Subsequent elaboration of this mol­ecular core has led to the development of (3a*SR*,10*RS*,10a*RS*)-2-(4-iodo­phen­yl)-1-oxo-5-tosyl-1,2,3,3a,4,5,10,10a-octa­hydro­pyrrolo­[3,4-*b*]carbazole-10-carb­oxy­lic acid ethanol solvent (**1**), a new and promising representative of the series. The synthetic approach to the structures of such a type requires elaboration and an efficient and general method for constructing diverse polycycles possessing the iso­indole core. The intra­molecular Diels–Alder reaction of vinyl­arenes (IMDAV) reaction represents a highly efficient strategy, enabling single-step preparation of iso­indole derivatives annulated with various carbo- and heterocyclic frameworks (Krishna *et al.*, 2022 ▸; Yakovleva *et al.*, 2024 ▸). Moreover, non-covalent bond donor or acceptor attached N-compounds are of inter­est due to their high solubility in polar solvents, functional properties, photoactivity in the solid state, coordination ability, high thermal and oxidative stability, *etc*. (Gurbanov *et al.*, 2018 ▸, 2023 ▸; Maharramov *et al.*, 2010 ▸, 2011 ▸; Pronina *et al.*, 2024 ▸). Functionalization of N-containing compounds with –COOH, –SO_3_H, *etc*. groups can improve catalytic activity and other properties (Burkin *et al.*, 2024 ▸; Mahmudov *et al.*, 2021 ▸, 2023 ▸).
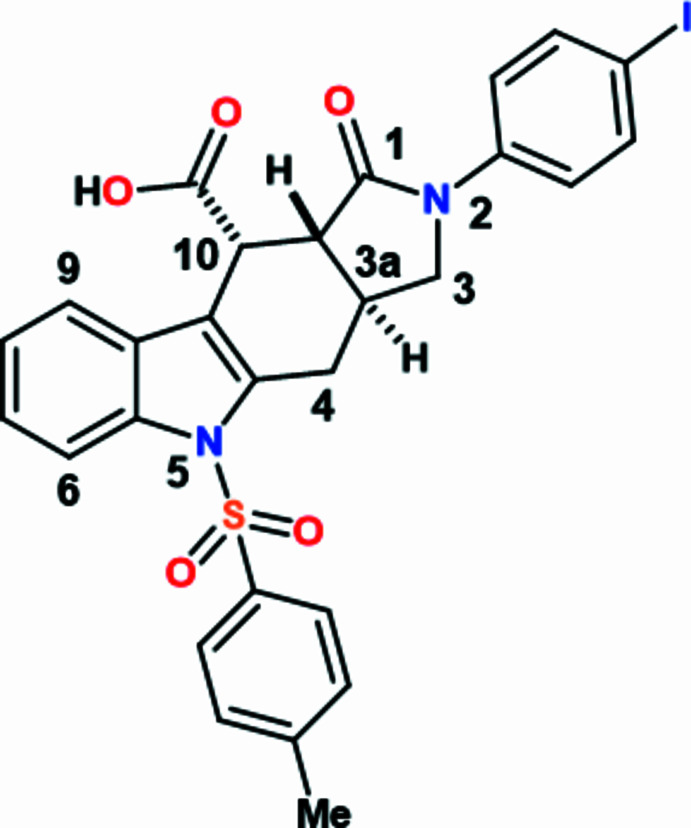


**Figure 1:**
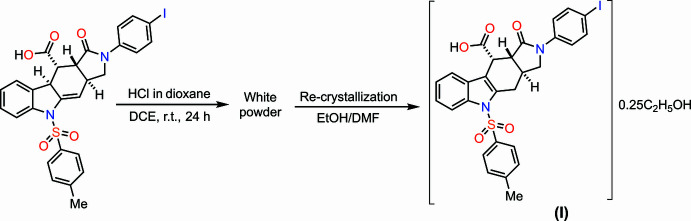
Reaction scheme to obtain the title compound (**I**).

**Figure 2:**
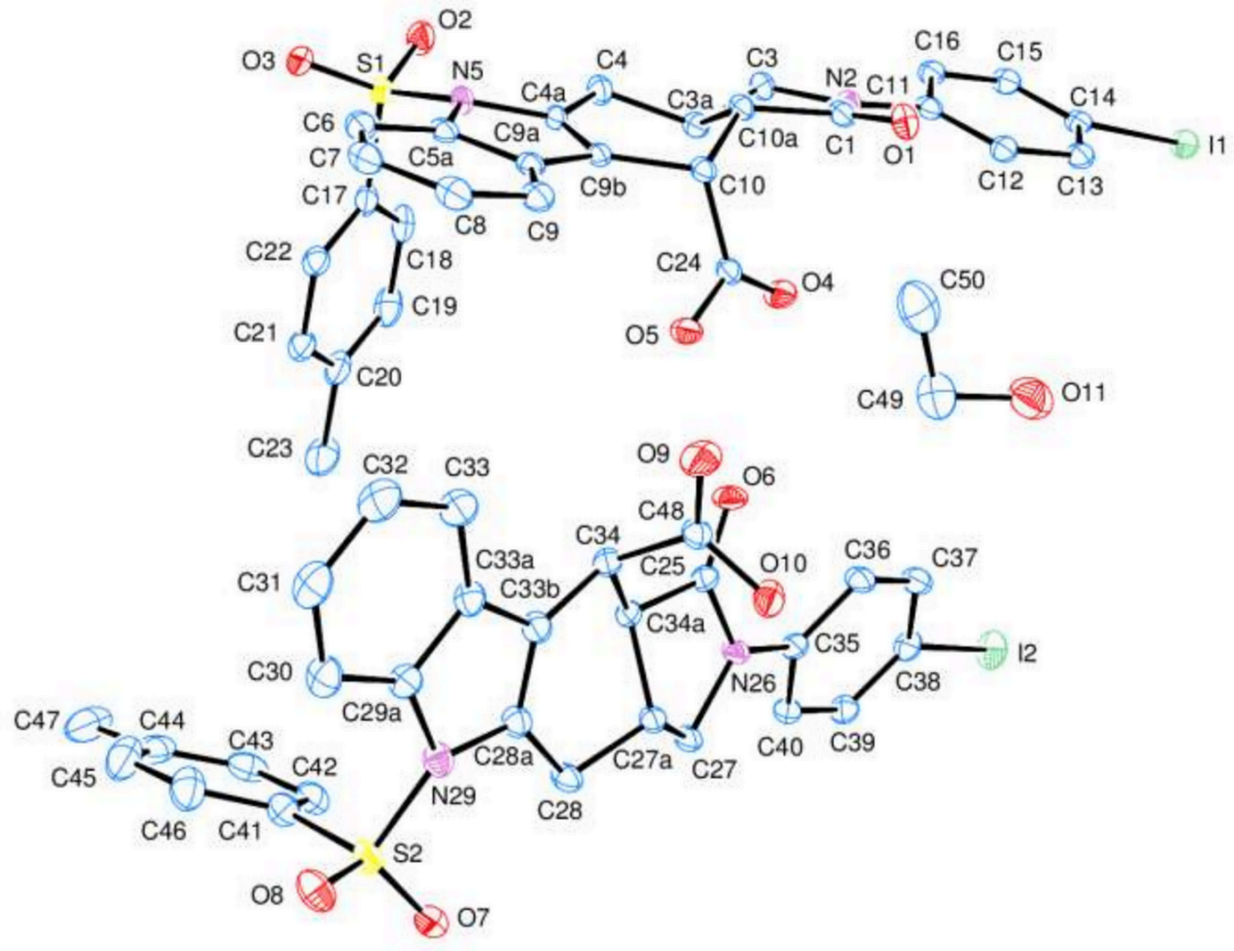
The asymmetric unit of the title compound (**I**) with the atom-numbering scheme and 50% probability ellipsoids, where the upper and lower mol­ecules are named as mol­ecules *A* and *B*, respectively. H atoms have been omitted for clarity.

In a continuation of our investigations into the properties of iso­indole­carb­oxy­lic acids previously synthesized from corresponding indolyl­allyl­amines *via* the IMDAV reaction (Shelukho *et al.*, 2025 ▸; Zubkov *et al.*, 2016 ▸; Horak *et al.*, 2015 ▸), we developed a highly efficient preparative protocol for the aromatization of [4 + 2] cyclo­addition adducts. Special emphasis was placed on optimizing the reaction conditions, leading to the identification of the most effective procedure involving acid-catalyzed isomerization in 1,2-di­chloro­ethane using an equimolar amount of hydrogen chloride in dioxane (Fig. 1 ▸). The selected conditions ensure qu­anti­tative conversion of the starting materials. Moreover, the developed protocol enables complete aromatizaion of certain mixtures of non-aromatic and aromatic adducts, facilitating structural elucidation of the resulting acids. The target product was isolated in 92% yield as a white crystalline solid. For unambiguous structural confirmation and verification of the degree of aromacity, single crystals were grown by controlled slow evaporation from an ethanol–DMF mixture. X-ray diffraction analysis conclusively confirmed complete aromatization of the polycyclic system, which is consistent with NMR spectroscopic data. The developed method demonstrates excellent reproducibility and can be successfully applied to the aromatization of structurally related analogues. Herein, we report the synthesis, mol­ecular and crystal structures together with the Hirshfeld surface analysis of the title compound (**I**)( Fig. 2 ▸).

## 2. Structural commentary

The asymmetric unit of the title compound (**I**) contains two crystallographically independent mol­ecules (*A* and *B*) and one ethanol solvent mol­ecule (Fig. 2 ▸). In mol­ecules *A* and *B*, the essentially planar *A* (C11–C16), *D* (N5/C4*A*/C5*A*/C9*A*/C9*B*), *E* (C5*A*/C6–C9/C9*A*) (in *A*), and *F* (C17–C22) and *G* (C35–C40), *J* (N29/C28*A*/C29*A*/C33*A*/C33*B*), *K* (C29*A*/C30–C33/C33*A*) and *L* (C41–C46)] (in *B*) rings are oriented at dihedral angles *A*/*D* = 5.56 (9)°, *A*/*E* = 7.69 (8)°, *A*/*F* = 82.16 (7)°, *D*/*E* = 2.80 (9)°, *D*/*F* = 87.21 (8)°, *E*/*F* = 87.98 (8)°, and *G*/*J* = 17.03 (9)°, *G*/*K* = 15.76 (8)°, *G*/*L* = 70.85 (8)°, *J*/*K* = 2.08 (8)°, *J*/*L* = 87.33 (9)°, *K*/*L* = 85.63 (8)°. It is clear that the *A*/*D* and *A*/*E* dihedral angles in mol­ecule *A* are much narrower than the corresponding ones (*G*/*J* and *G*/*K*) in mol­ecule *B*. On the other hand, the *A*/*F* dihedral angle in mol­ecule *A* is considerably enlarged with respect to the corresponding one (*G*/*L*) in mol­ecule *B* due to the intra- and inter­molecular C—H⋯O and O—H⋯O hydrogen bonds (Table 1 ▸). The *B* (N2/C1/C3/C3*A*/C10*A* and *C* (C3A/C4/C4A/C9B/C10/C10A) (in mol­ecule *A*) and *H* (N26/C25/C27/C27*A*/C34*A*) and *I* (C27*A*/C28/C28*A*/C33B/C34/C34*A*) (in mol­ecule *B*) rings exhibit envelope conformations, where atoms C3*A*, C10*A*, C27*A* and C34*A* occupy the flap positions, displaced by −0.534 (3), 0.698 (2), −0.600 (3) and 0.652 (3) Å away from the best least-squares planes of the other atoms. The O4—C24 [1.211 (3) Å], O5—C24 [1.328 (3) Å], O9—C48 [1.205 (3) Å] and O10—C48 [1.320 (3) Å] distances in the carb­oxy­lic acid moieties indicate localized single and double bonds rather than delocalized bonding arrangements. The O4—C24—O5 [123.9 (2)°] and O9—C48—O10 [123.9 (2)°] bond angles are increased with respect to that in a free acid (122.2°, Sim *et al.*, 1955 ▸) and may be compared with the corresponding values of 124.27 (17)° in di­aqua­bis­(2-bromo­benzoato-O)bis­(nicotinamide-κ*N*^1^)zinc(II) (Hökelek *et al.*, 2009 ▸), 126.3 (3)° in *trans*-di­aqua­bis­(*N*,*N*-di­ethyl­nicotinamide-κ*N*^1^)bis­(4-nitro­benzoato-κ*O*)copper(II) (Hökelek *et al.*, 1997 ▸) and 122.55 (12)° in methyl 2-oxo-1-(prop-2-yn­yl)-1,2-di­hydro­quinoline-4-carboxyl­ate (El-Mrabet *et al.*, 2023 ▸). No unusual bond distances or inter­bond angles are observed in (**I**).

## 3. Supra­molecular features

**Figure 3:**
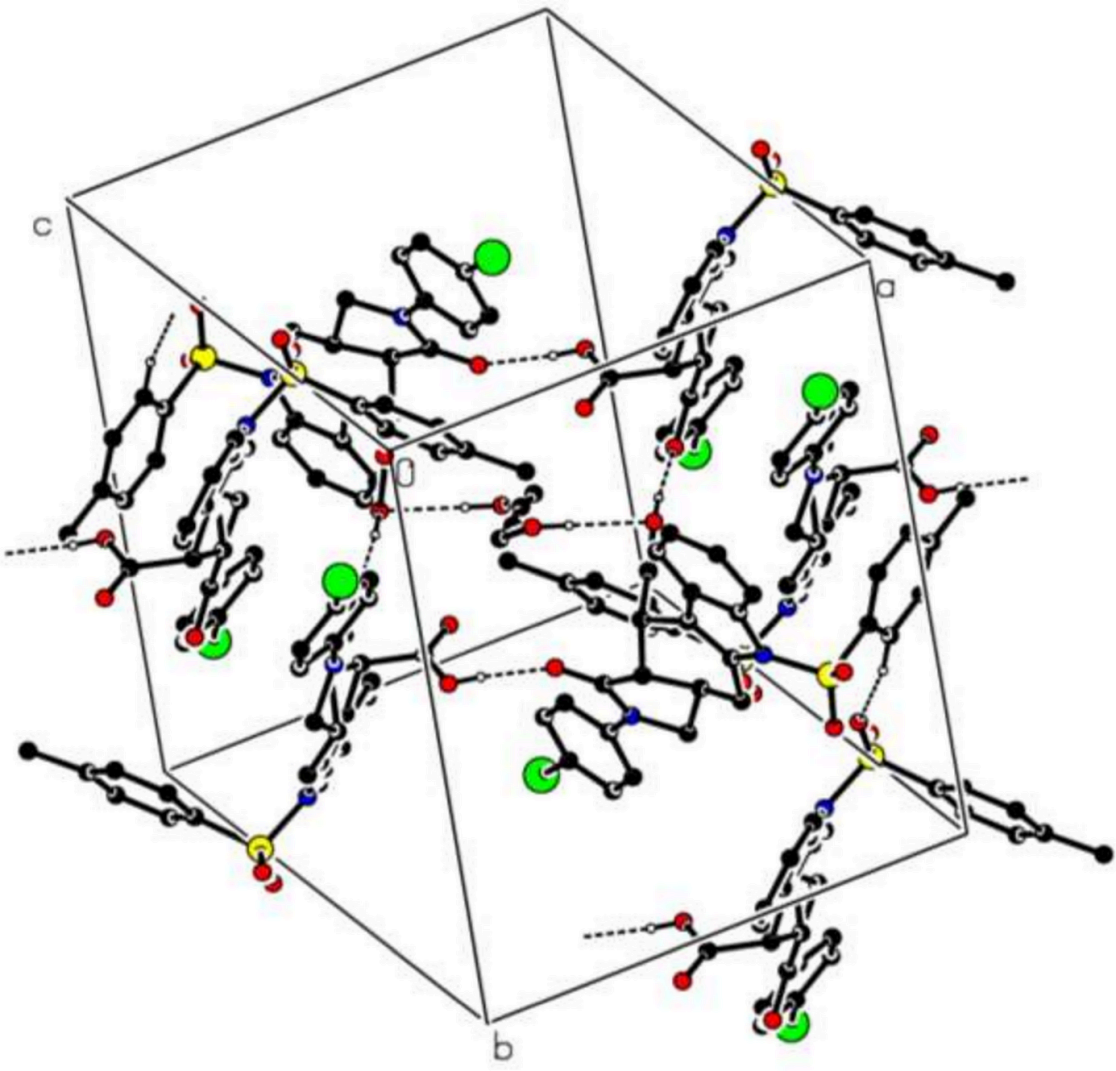
A partial packing diagram for the title compound (**I**). The O—H⋯O and C—H⋯O hydrogen bonds are shown as dashed lines. H atoms not involved in these inter­actions have been omitted for clarity.

In the crystal, O—H⋯O and C—H⋯O hydrogen bonds (Table 1 ▸) link the mol­ecules into a three-dimensional architecture, enclosing 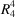
(23) ring motifs (Etter *et al.*, 1990 ▸) (Fig. 3 ▸). The O5—H5*O*⋯O6 hydrogen bond links the two independent mol­ecules in the asymmetric unit while the C18—H18⋯O8 and O10—H10*O*⋯O5 hydrogen bonds as well as the O11—H11O⋯O5 hydrogen bond between the solvent mol­ecule and mol­ecule *A* contribute to the supra­molecular behaviour. Further the C—H⋯π(ring) inter­actions and the π–π inter­actions between the *F* [centroid-to-centroid distance = 3.6818 (15) Å, α = 0.02 (12)° and slippage = 1.084 Å] and *G* rings of adjacent mol­ecules [centroid-to-centroid distance = 3.6747 (14) Å, α = 0.00 (12)° and slippage = 1.496 Å], and the *D* and *E* rings [centroid-to-centroid distance = 3.7727 (15) Å, α = 2.79 (13)° and slippage = 1.355 Å] help to consolidate the packing.

## 4. Hirshfeld surface analysis

**Figure 4:**
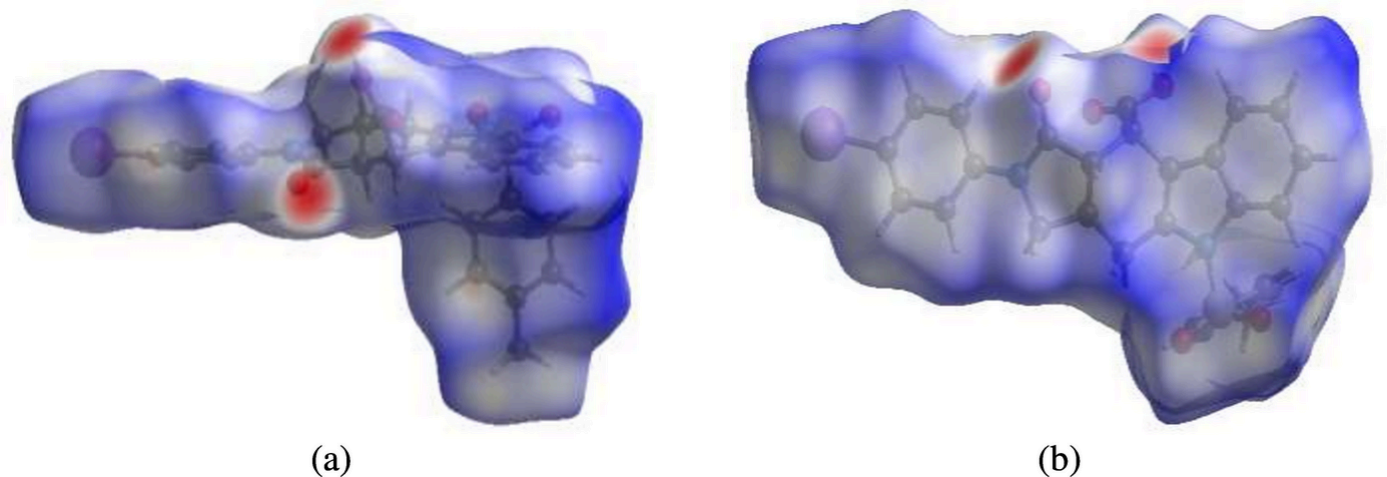
Views of the three-dimensional Hirshfeld surfaces for mol­ecules (*a*) *A* and (*b*) *B* plotted over *d*_norm_.

**Figure 5:**
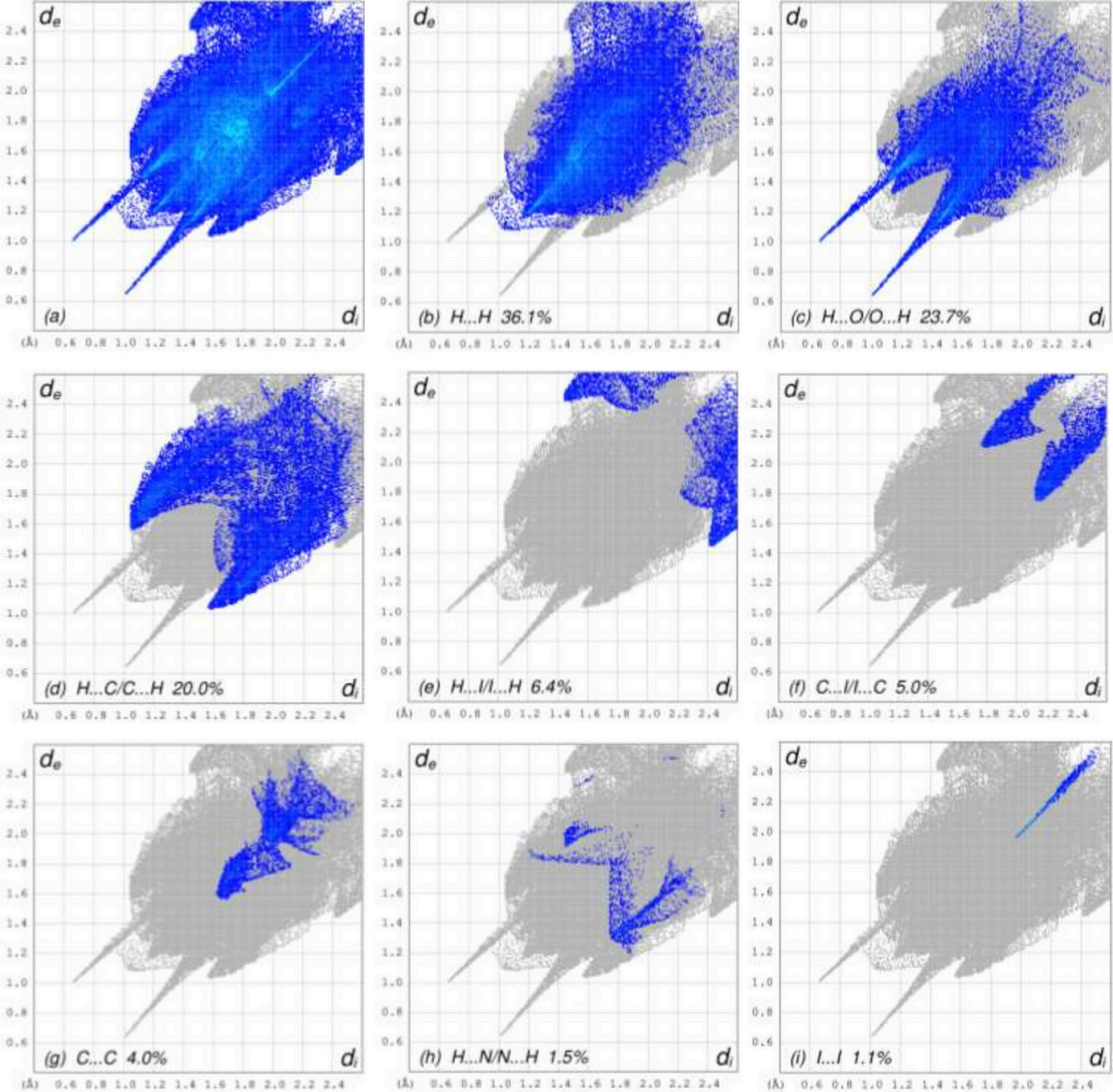
The full two-dimensional fingerprint plots for mol­ecule *A*, showing (*a*) all inter­actions, and delineated into (*b*) H⋯H, (*c*) H⋯O/O⋯H, (*d*) H⋯C/C⋯H, (*e*) H⋯I/I⋯H, (*f*) C⋯I/I⋯C, (*g*) C⋯C, (*h*) H⋯N/N⋯H, (i) I⋯I, (*j*) O⋯O, (*k*) C⋯O/O⋯C, (*l*) O⋯I/I⋯O, (*m*) N⋯O/O⋯N and (*n*) C⋯N/N⋯C inter­actions. The *d*_i_ and *d*_e_ values are the closest inter­nal and external distances (in Å) from given points on the Hirshfeld surface.

To visualize the inter­molecular inter­actions in the crystal of title compound (**I**), a Hirshfeld surface (HS) analysis was carried out using *Crystal Explorer 17.5* (Spackman *et al.*, 2021 ▸). In the HS plotted over *d*_norm_ (Fig. 4 ▸*a* and *b*), the contact distances equal, shorter and longer with respect to the sum of van der Waals radii are shown the white, red and blue colours, respectively. According to the two-dimensional fingerprint plots, H⋯H, H⋯O/O⋯H, H⋯C/C⋯H and H⋯I/I⋯H contacts make the most important contributions to the HS (Figs. 5 ▸ and 6 ▸), and they have significant differences due to the different numbers and values of the close contacts (see supporting information).

## 5. Synthesis and crystallization

Anequimolar amount of HCl in dioxane (5.0 mol L^−1^; 0.250 mmol, 0.0045 mL) was added to a suspension of the starting material (3a*RS*,9b*RS*,10*RS*,10a*RS*)-2-(4-iodo­phen­yl)-5-[(4-methyl­phen­yl)sulfon­yl]-1-oxo-1,2,3,3a,4,5,10,10a-octa­hydro­pyrrolo­[3,4-*b*]carbozole-10-carb­oxy­lic acid (0.250 mmol, 0.13 g) in DCE (10 mL). The resulting mixture was stirred at r.t. for 24 h. The resulting precipitate was filtered off, washed with diethyl ether (5 mL), and air-dried to afford the target product (3a*S*,10*R*,10a*R*)-2-(4-iodo­phen­yl)-1-oxo-5-tosyl-1,2,3,3a,4,5,10,10a-octa­hydro­pyrrolo­[3,4-*b*]carbazole-10-carb­oxy­lic acid as white powder (0.23 mmol, 92%). Single crystals suitable for X-ray diffraction were obtained by slow evaporation of a mixture of ethanol and DMF. Yield 92%, 0.12 g; m.p. 538–543 K. ^1^H NMR (700 MHz, DMSO-*d_6_*, 298 K) δ 12.81 (*br. s*, 1H, CO_2_H), 8.05 (*d*, *J* = 8.3 Hz, 1H, H-Ar), 7.83 (*m*, *J* = 8.3 Hz, 2H, H-Ar), 7.78–7.71 (*m*, 3H, H-Ar), 7.54 (*m*, 2H, H-Ar), 7.40–7.25 (*m*, 5H, H-Ar), 4.14 (*d*, *J* = 4.0 Hz, 1H, H-10), 4.08 (*t*, *J* = 8.0 Hz, 1H, H-3), 3.80 (*t*, *J* = 10.0 Hz, 1H, H-10a), 3.59 (*dd*, *J* = 16.9, 5.0 Hz, 1H, H-3), 3.05–2.91 (*m*, 2H, H-3a, H-4), 2.32 (*s*, 3H, H-CH_3_) ppm ^13^C NMR (176.1 MHz, DMSO-*d_6_*, 298 K) δ 172.8, 172.6, 146.0, 140.0, 137.8 (2C) 136.7, 136.0, 135.1, 130.8 (2C), 129.0, 128.7 (2C), 126.9 (2C), 125.8, 125.0, 121.5, 116.4, 114.2, 88.1, 51.6, 47.1, 37.2, 32.1, 28.3, 21.5 ppm. MS (ESI): *m*/*z* = 627 [*M* + H]^+^. Analysis calculated for C_28_H_23_O_5_IN_2_S: C, 53.68; H, 3.70; O, 12.77; N, 4.47; S, 5.12; found: C, 53.51; H, 3.52; O, 12.62; N, 4.52; S, 5.32.

## 6. Refinement

Crystal data, data collection and structure refinement details are summarized in Table 2 ▸. The OH hydrogen atoms were located in a difference-Fourier map, and refined isotropically. The C-bound hydrogen-atom positions were calculated geometrically at distances of 1.00 (for methine CH), 0.95 (for aromatic CH), 0.99 (for methyl­ene CH) and 0.98 Å (for CH_3_) and refined using a riding model by applying the constraint *U*_iso_(H) = *k* × *U*_eq_(C), where *k* = 1.5 for methyl H atoms and k = 1.2 for the other H atoms. The ethanol solvent mol­ecule is disordered relative to the inversion center. To fit its geometry to the ideal theoretical one, upon refinement the three intra­molecular distances were fixed with the accuracy of 0.003 Å: O11—C49 = 1.430 (3) Å, C49—C50 = 1.525 (3) Å and O11⋯C50 = 2.450 (3) Å. The hydrogen atom of the OH group was objectively localized in the difference-Fourier maps and refined within the riding model with fixed positional (at 0.90 Å) and isotropic displacement parameters [*U*_iso_(H) = 1.5*U*_eq_(O)]. The other hydrogen atoms in this mol­ecule were placed in calculated positions and refined within the riding model with fixed isotropic displacement parameters [*U*_iso_(H) = 1.5*U*_eq_(C) for the CH_3_ group and 1.2*U*_eq_(C) for the CH_2_ group].

## Supplementary Material

Crystal structure: contains datablock(s) I, global. DOI: 10.1107/S2056989025011582/vu2015sup1.cif

Structure factors: contains datablock(s) I. DOI: 10.1107/S2056989025011582/vu2015Isup2.hkl

Supplementary tables. DOI: 10.1107/S2056989025011582/vu2015sup3.pdf

CCDC reference: 2518350

Additional supporting information:  crystallographic information; 3D view; checkCIF report

## Figures and Tables

**Figure 6:**
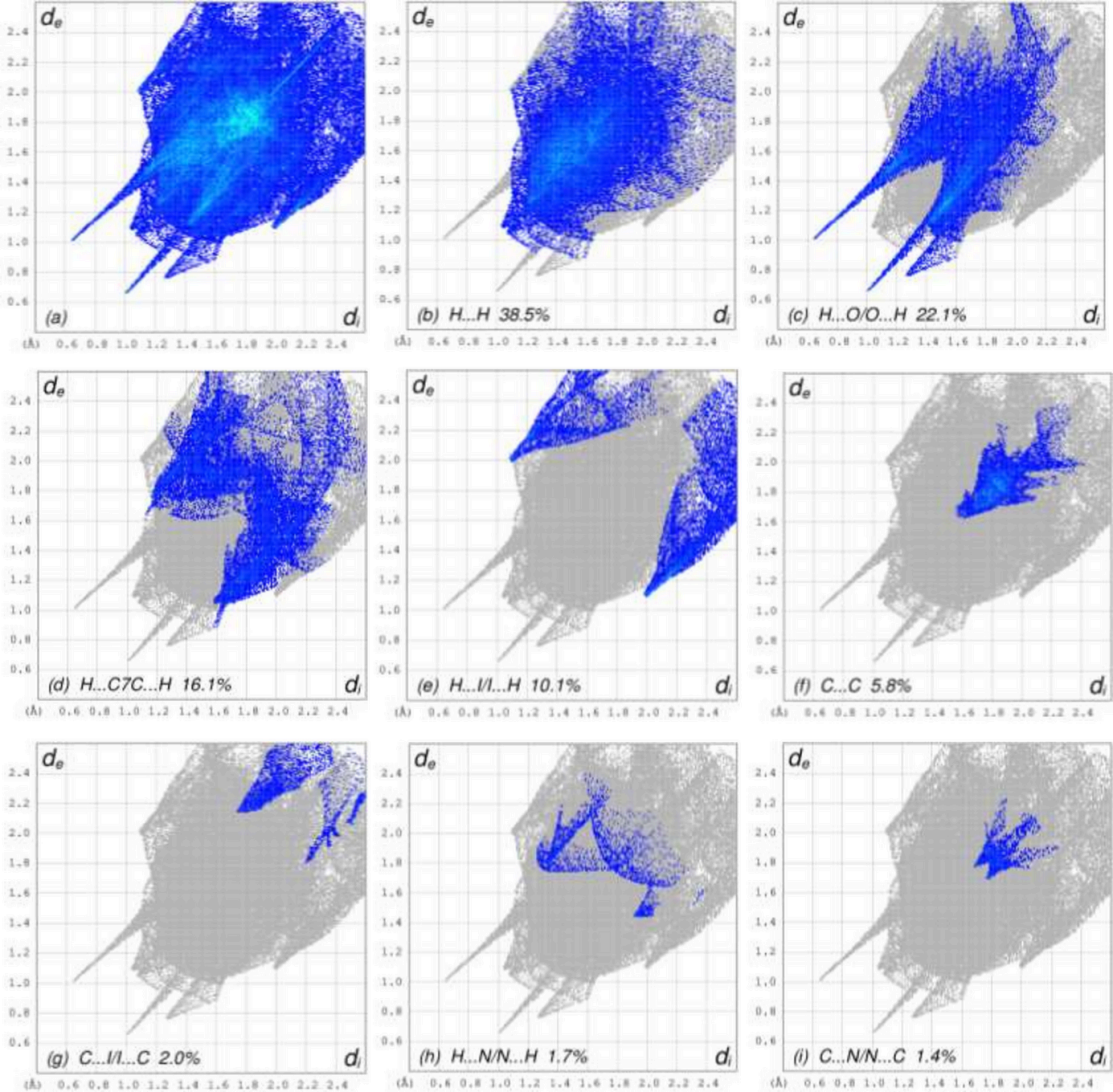
The full two-dimensional fingerprint plots for mol­ecule *B*, showing (*a*) all inter­actions, and delineated into (*b*) H⋯H, (*c*) H⋯O/O⋯H, (*d*) H⋯C/C⋯H, (*e*) H⋯I/I⋯H, (*f*) C⋯C, (*g*) C⋯I/I⋯C, (*h*) H⋯N/N⋯H, (i) C⋯N/N⋯C, (*j*) I⋯I, (*k*) C⋯O/O⋯C and (*l*) O⋯O inter­actions.

**Table 1:** Hydrogen-bond geometry (Å, °) *Cg*6, *Cg*8 and *Cg*12 are the centroids of the C5*A*/C6–C9/C9*A*, C17–C22 and C41–C46 rings, respectively.

*D*—H⋯*A*	*D*—H	H⋯*A*	*D*⋯*A*	*D*—H⋯*A*
O5—H5*O*⋯O6	0.85 (4)	1.80 (4)	2.640 (2)	174 (4)
C3*A*—H3*C*⋯O4	1.00	2.44	3.066 (3)	120
C6—H6⋯O3	0.95	2.32	2.913 (4)	120
C12—H12⋯O1	0.95	2.23	2.838 (3)	121
C18—H18⋯O8^i^	0.95	2.45	3.298 (3)	148
O10—H10*O*⋯O1^ii^	0.78 (5)	1.87 (5)	2.623 (3)	163 (5)
C27*A*—H27*C*⋯O10	1.00	2.35	3.011 (3)	123
C30—H30⋯O8	0.95	2.40	2.980 (4)	119
C36—H36⋯O6	0.95	2.35	2.895 (3)	116
O11—H11*O*⋯O5^ii^	0.90	2.12	3.024 (6)	177
C27—H27*A*⋯*Cg*8^iii^	0.99	2.87	3.729 (3)	145
C37—H37⋯*Cg*12^iv^	0.95	2.93	3.776 (3)	148
C39—H39⋯*Cg*6^iii^	0.95	2.70	3.526 (3)	145

Symmetry codes: (i) 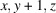; (ii) 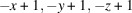; (iii) 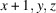; (iv) 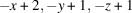.

**Table 2:** Experimental details

Crystal data	
Chemical formula	4C_28_H_23_IN_2_O_5_S·C_2_H_6_O
*M*_r_	2551.84
Crystal system, space group	Triclinic, *P* 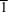
Temperature (K)	100
*a*, *b*, *c* (Å)	12.39864 (18), 14.7576 (2), 15.05254 (16)
α, β, γ (°)	100.860 (1), 103.3627 (10), 98.5545 (12)
*V* (Å^3^)	2577.83 (6)
*Z*	1
Radiation type	Cu *K*α
μ (mm^−1^)	10.89
Crystal size (mm)	0.21 × 0.09 × 0.09

Data collection	
Diffractometer	Rigaku XtaLAB Synergy-S, HyPix-6000HE area-detector
Absorption correction	Multi-scan (*CrysAlis PRO*; Rigaku OD, 2025 ▸)
*T*_min_, *T*_max_	0.307, 1.000
No. of measured, independent and observed [*I* > 2σ(*I*)] reflections	57678, 11116, 10276
*R*_int_	0.063
(sin θ/λ)_max_ (Å^−1^)	0.639

Refinement	
*R*[*F*^2^ > 2σ(*F*^2^)], *wR*(*F*^2^), *S*	0.036, 0.103, 1.08
No. of reflections	11116
No. of parameters	703
No. of restraints	3
H-atom treatment	H atoms treated by a mixture of independent and constrained refinement
Δρ_max_, Δρ_min_ (e Å^−3^)	0.73, −1.49

Computer programs: *CrysAlis PRO* (Rigaku OD, 2025 ▸), *SHELXT* (Sheldrick, 2015*a* ▸), *SHELXL* (Sheldrick, 2015*b* ▸) and *SHELXTL* (Sheldrick, 2008 ▸).

## supplementary crystallographic information

### (3aSR,10RS,10aRS)-2-(4-Iodophenyl)-1-oxo-5-tosyl-1,2,3,3a,4,5,10,10a-octahydropyrrolo[3,4-b]carbazole-10-carboxylic acid–ethanol (4/1) Crystal data

**Table:**

4C_28_H_23_IN_2_O_5_S·C_2_H_6_O	*Z* = 1
*M**_r_* = 2551.84	*F*(000) = 1282
Triclinic, *P*1	*D*_x_ = 1.644 Mg m^−^^3^
*a* = 12.39864 (18) Å	Cu *K*α radiation, λ = 1.54184 Å
*b* = 14.7576 (2) Å	Cell parameters from 34693 reflections
*c* = 15.05254 (16) Å	θ = 3.1–79.7°
α = 100.860 (1)°	µ = 10.89 mm^−^^1^
β = 103.3627 (10)°	*T* = 100 K
γ = 98.5545 (12)°	Prism, colourless
*V* = 2577.83 (6) Å^3^	0.21 × 0.09 × 0.09 mm

### (3aSR,10RS,10aRS)-2-(4-Iodophenyl)-1-oxo-5-tosyl-1,2,3,3a,4,5,10,10a-octahydropyrrolo[3,4-b]carbazole-10-carboxylic acid–ethanol (4/1) Data collection

**Table:**

Rigaku XtaLAB Synergy-S, HyPix-6000HE area-detector diffractometer	10276 reflections with *I* > 2σ(*I*)
Radiation source: micro-focus sealed X-ray tube	*R*_int_ = 0.063
φ and ω scans	θ_max_ = 80.1°, θ_min_ = 3.1°
Absorption correction: multi-scan (CrysAlisPro; Rigaku OD, 2025)	*h* = −15→15
*T*_min_ = 0.307, *T*_max_ = 1.000	*k* = −18→18
57678 measured reflections	*l* = −15→18
11116 independent reflections	

### (3aSR,10RS,10aRS)-2-(4-Iodophenyl)-1-oxo-5-tosyl-1,2,3,3a,4,5,10,10a-octahydropyrrolo[3,4-b]carbazole-10-carboxylic acid–ethanol (4/1) Refinement

**Table:**

Refinement on *F*^2^	Primary atom site location: difference Fourier map
Least-squares matrix: full	Secondary atom site location: difference Fourier map
*R*[*F*^2^ > 2σ(*F*^2^)] = 0.036	Hydrogen site location: mixed
*wR*(*F*^2^) = 0.103	H atoms treated by a mixture of independent and constrained refinement
*S* = 1.08	*w* = 1/[σ^2^(*F*_o_^2^) + (0.0678*P*)^2^ + 0.6004*P*] where *P* = (*F*_o_^2^ + 2*F*_c_^2^)/3
11116 reflections	(Δ/σ)_max_ = 0.001
703 parameters	Δρ_max_ = 0.73 e Å^−^^3^
3 restraints	Δρ_min_ = −1.49 e Å^−^^3^

### (3aSR,10RS,10aRS)-2-(4-Iodophenyl)-1-oxo-5-tosyl-1,2,3,3a,4,5,10,10a-octahydropyrrolo[3,4-b]carbazole-10-carboxylic acid–ethanol (4/1) Special details

**Table:**

Geometry. All esds (except the esd in the dihedral angle between two l.s. planes) are estimated using the full covariance matrix. The cell esds are taken into account individually in the estimation of esds in distances, angles and torsion angles; correlations between esds in cell parameters are only used when they are defined by crystal symmetry. An approximate (isotropic) treatment of cell esds is used for estimating esds involving l.s. planes.

### (3aSR,10RS,10aRS)-2-(4-Iodophenyl)-1-oxo-5-tosyl-1,2,3,3a,4,5,10,10a-octahydropyrrolo[3,4-b]carbazole-10-carboxylic acid–ethanol (4/1) Fractional atomic coordinates and isotropic or equivalent isotropic displacement parameters (Å^2^)

**Table:**

	*x*	*y*	*z*	*U*_iso_*/*U*_eq_	Occ. (<1)
I1	0.62834 (2)	1.12895 (2)	0.80589 (2)	0.02282 (6)	
S1	0.80588 (5)	0.67569 (4)	0.03768 (4)	0.01867 (12)	
O1	0.48320 (15)	0.72592 (13)	0.42547 (13)	0.0207 (3)	
O2	0.80596 (18)	0.77433 (14)	0.05512 (13)	0.0253 (4)	
O3	0.78203 (17)	0.62072 (14)	−0.05633 (12)	0.0231 (4)	
O4	0.71115 (16)	0.65374 (13)	0.45052 (12)	0.0216 (4)	
O5	0.68875 (16)	0.51002 (12)	0.35881 (12)	0.0199 (3)	
H5O	0.733 (3)	0.505 (3)	0.409 (3)	0.030*	
C1	0.5560 (2)	0.76524 (17)	0.39477 (17)	0.0170 (4)	
N2	0.61722 (17)	0.85528 (15)	0.42748 (14)	0.0164 (4)	
C3	0.6875 (2)	0.88120 (17)	0.36464 (17)	0.0176 (4)	
H3A	0.762066	0.920650	0.401039	0.021*	
H3B	0.648655	0.914951	0.319948	0.021*	
C3A	0.6994 (2)	0.78430 (17)	0.31391 (16)	0.0160 (4)	
H3C	0.760587	0.763425	0.356432	0.019*	
C4	0.7227 (2)	0.77192 (17)	0.21743 (17)	0.0188 (5)	
H4A	0.675967	0.806082	0.178144	0.023*	
H4B	0.803421	0.797074	0.223943	0.023*	
C4A	0.6930 (2)	0.66801 (17)	0.17307 (16)	0.0158 (4)	
N5	0.70751 (18)	0.62733 (15)	0.08390 (14)	0.0171 (4)	
C5A	0.66157 (19)	0.52894 (17)	0.06230 (16)	0.0168 (4)	
C6	0.6519 (2)	0.4588 (2)	−0.01715 (18)	0.0225 (5)	
H6	0.682630	0.471500	−0.066796	0.027*	
C7	0.5946 (2)	0.36878 (19)	−0.01997 (19)	0.0236 (5)	
H7	0.586150	0.319238	−0.073055	0.028*	
C8	0.5491 (2)	0.34935 (19)	0.05320 (19)	0.0237 (5)	
H8	0.510531	0.287373	0.048823	0.028*	
C9	0.5602 (2)	0.41990 (18)	0.13175 (18)	0.0204 (5)	
H9	0.529871	0.406948	0.181529	0.025*	
C9A	0.6169 (2)	0.51039 (17)	0.13617 (16)	0.0164 (4)	
C9B	0.63787 (19)	0.59880 (17)	0.20452 (16)	0.0153 (4)	
C10	0.59346 (19)	0.61824 (16)	0.29041 (16)	0.0150 (4)	
H10	0.515855	0.579106	0.276718	0.018*	
C10A	0.58696 (19)	0.72211 (16)	0.30752 (16)	0.0151 (4)	
H10A	0.529775	0.730572	0.252403	0.018*	
C11	0.6132 (2)	0.91830 (17)	0.50950 (17)	0.0170 (4)	
C12	0.5742 (2)	0.88460 (18)	0.57877 (17)	0.0178 (4)	
H12	0.546902	0.818977	0.569699	0.021*	
C13	0.5747 (2)	0.94634 (18)	0.66199 (17)	0.0187 (5)	
H13	0.547100	0.922894	0.708867	0.022*	
C14	0.6157 (2)	1.04195 (18)	0.67529 (16)	0.0176 (4)	
C15	0.6539 (2)	1.07669 (17)	0.60631 (17)	0.0189 (4)	
H15	0.681424	1.142353	0.615897	0.023*	
C16	0.6520 (2)	1.01539 (18)	0.52263 (17)	0.0184 (4)	
H16	0.676817	1.039398	0.474894	0.022*	
C17	0.9340 (2)	0.65741 (18)	0.10353 (17)	0.0194 (5)	
C18	1.0085 (2)	0.7318 (2)	0.17129 (18)	0.0240 (5)	
H18	0.990255	0.792446	0.183245	0.029*	
C19	1.1100 (2)	0.7153 (2)	0.22092 (19)	0.0285 (6)	
H19	1.161068	0.765289	0.267946	0.034*	
C20	1.1388 (2)	0.6267 (2)	0.20321 (18)	0.0255 (5)	
C21	1.0614 (2)	0.5536 (2)	0.13549 (17)	0.0228 (5)	
H21	1.079221	0.492850	0.123223	0.027*	
C22	0.9592 (2)	0.56828 (18)	0.08596 (17)	0.0195 (5)	
H22	0.906898	0.517910	0.040420	0.023*	
C23	1.2493 (3)	0.6095 (3)	0.2567 (2)	0.0369 (7)	
H23A	1.311561	0.657568	0.254311	0.055*	
H23B	1.259680	0.546983	0.228385	0.055*	
H23C	1.248703	0.612568	0.322162	0.055*	
C24	0.67064 (19)	0.59732 (17)	0.37605 (17)	0.0162 (4)	
I2	1.11860 (2)	0.79531 (2)	0.96304 (2)	0.02919 (7)	
S2	1.03004 (5)	0.02919 (4)	0.35060 (4)	0.02059 (12)	
O6	0.83670 (16)	0.49561 (14)	0.51016 (13)	0.0222 (4)	
O7	1.08688 (17)	0.05066 (14)	0.44799 (14)	0.0244 (4)	
O8	0.98904 (18)	−0.06680 (14)	0.29942 (15)	0.0292 (4)	
O9	0.59404 (17)	0.28231 (18)	0.36431 (15)	0.0323 (5)	
O10	0.69260 (16)	0.27402 (16)	0.50547 (14)	0.0270 (4)	
H10O	0.633 (4)	0.270 (3)	0.515 (3)	0.041*	
C25	0.8940 (2)	0.43778 (18)	0.53221 (16)	0.0173 (4)	
N26	0.96981 (17)	0.44835 (15)	0.61642 (14)	0.0172 (4)	
C27	1.0284 (2)	0.36768 (18)	0.61789 (17)	0.0174 (4)	
H27A	1.040691	0.350718	0.679523	0.021*	
H27B	1.102056	0.381589	0.603160	0.021*	
C27A	0.94458 (19)	0.28986 (17)	0.54076 (16)	0.0160 (4)	
H27C	0.881869	0.263545	0.565889	0.019*	
C28	0.9920 (2)	0.20916 (18)	0.49494 (17)	0.0195 (5)	
H28A	1.068048	0.233262	0.488440	0.023*	
H28B	0.998713	0.162100	0.533841	0.023*	
C28A	0.9115 (2)	0.16489 (18)	0.39981 (18)	0.0189 (5)	
N29	0.91600 (19)	0.07836 (16)	0.34149 (15)	0.0203 (4)	
C29A	0.8285 (2)	0.06166 (18)	0.25696 (18)	0.0202 (5)	
C30	0.7991 (3)	−0.00993 (19)	0.17547 (19)	0.0258 (5)	
H30	0.842140	−0.057612	0.167956	0.031*	
C31	0.7042 (3)	−0.0087 (2)	0.1058 (2)	0.0297 (6)	
H31	0.680860	−0.057741	0.050328	0.036*	
C32	0.6421 (3)	0.0627 (2)	0.1148 (2)	0.0315 (6)	
H32	0.578057	0.061636	0.065332	0.038*	
C33	0.6727 (2)	0.1349 (2)	0.19475 (19)	0.0261 (5)	
H33	0.630632	0.183460	0.200856	0.031*	
C33A	0.7672 (2)	0.13445 (18)	0.26649 (18)	0.0202 (5)	
C33B	0.8223 (2)	0.19868 (18)	0.35581 (17)	0.0188 (5)	
C34	0.7948 (2)	0.29292 (18)	0.39186 (17)	0.0172 (4)	
H34	0.787515	0.327079	0.340034	0.021*	
C34A	0.8990 (2)	0.34667 (17)	0.47004 (16)	0.0157 (4)	
H34A	0.960130	0.362053	0.438874	0.019*	
C35	1.0035 (2)	0.53027 (17)	0.69079 (16)	0.0170 (4)	
C36	0.9290 (2)	0.59036 (19)	0.70616 (17)	0.0213 (5)	
H36	0.855650	0.578861	0.664305	0.026*	
C37	0.9630 (2)	0.6671 (2)	0.78305 (18)	0.0240 (5)	
H37	0.912493	0.707873	0.793924	0.029*	
C38	1.0705 (2)	0.68407 (19)	0.84388 (17)	0.0211 (5)	
C39	1.1455 (2)	0.62533 (19)	0.82786 (17)	0.0204 (5)	
H39	1.219376	0.637747	0.869066	0.025*	
C40	1.1119 (2)	0.54866 (19)	0.75158 (18)	0.0192 (5)	
H40	1.162940	0.508463	0.740621	0.023*	
C41	1.1164 (2)	0.0884 (2)	0.29294 (19)	0.0230 (5)	
C42	1.1868 (2)	0.1739 (2)	0.3408 (2)	0.0252 (5)	
H42	1.186170	0.201767	0.402835	0.030*	
C43	1.2587 (2)	0.2183 (2)	0.2964 (2)	0.0277 (6)	
H43	1.305753	0.277910	0.328102	0.033*	
C44	1.2629 (2)	0.1770 (2)	0.2064 (2)	0.0319 (6)	
C45	1.1895 (3)	0.0917 (3)	0.1590 (2)	0.0365 (7)	
H45	1.189889	0.063949	0.096786	0.044*	
C46	1.1158 (3)	0.0466 (2)	0.2013 (2)	0.0321 (6)	
H46	1.065884	−0.011500	0.168655	0.039*	
C47	1.3487 (3)	0.2237 (3)	0.1635 (3)	0.0414 (8)	
H47A	1.425207	0.223167	0.199541	0.062*	
H47B	1.340375	0.288878	0.164756	0.062*	
H47C	1.335981	0.189207	0.098423	0.062*	
C48	0.6826 (2)	0.28292 (18)	0.41848 (18)	0.0189 (5)	
O11	0.5545 (4)	0.5639 (4)	0.6475 (3)	0.0417 (11)	0.5
H11O	0.482501	0.543393	0.647764	0.063*	0.5
C49	0.5758 (5)	0.5140 (7)	0.5644 (3)	0.045 (2)	0.5
H49A	0.591186	0.451957	0.573371	0.055*	0.5
H49B	0.644154	0.549660	0.553980	0.055*	0.5
C50	0.4768 (5)	0.4993 (6)	0.4780 (3)	0.043 (2)	0.5
H50A	0.487326	0.452873	0.426491	0.065*	0.5
H50B	0.473377	0.559117	0.459375	0.065*	0.5
H50C	0.406148	0.476269	0.492649	0.065*	0.5

### (3aSR,10RS,10aRS)-2-(4-Iodophenyl)-1-oxo-5-tosyl-1,2,3,3a,4,5,10,10a-octahydropyrrolo[3,4-b]carbazole-10-carboxylic acid–ethanol (4/1) Atomic displacement parameters (Å^2^)

**Table:**

	*U*^11^	*U*^22^	*U*^33^	*U*^12^	*U*^13^	*U*^23^
I1	0.02824 (10)	0.02330 (10)	0.01369 (9)	0.00995 (7)	0.00207 (6)	−0.00291 (6)
S1	0.0247 (3)	0.0202 (3)	0.0135 (3)	0.0097 (2)	0.0067 (2)	0.0036 (2)
O1	0.0178 (8)	0.0213 (9)	0.0212 (8)	0.0018 (7)	0.0078 (7)	−0.0013 (7)
O2	0.0382 (11)	0.0208 (9)	0.0231 (9)	0.0113 (8)	0.0149 (8)	0.0075 (7)
O3	0.0306 (10)	0.0277 (9)	0.0136 (8)	0.0134 (8)	0.0067 (7)	0.0047 (7)
O4	0.0247 (9)	0.0214 (9)	0.0140 (8)	0.0069 (7)	−0.0015 (7)	−0.0005 (7)
O5	0.0239 (9)	0.0180 (8)	0.0153 (8)	0.0092 (7)	−0.0003 (7)	0.0010 (6)
C1	0.0150 (10)	0.0186 (11)	0.0162 (11)	0.0070 (9)	0.0022 (8)	0.0004 (9)
N2	0.0152 (9)	0.0168 (9)	0.0153 (9)	0.0044 (7)	0.0032 (7)	−0.0002 (7)
C3	0.0207 (11)	0.0148 (11)	0.0176 (11)	0.0059 (9)	0.0064 (9)	0.0011 (9)
C3A	0.0154 (10)	0.0151 (10)	0.0150 (11)	0.0041 (8)	0.0014 (8)	0.0000 (8)
C4	0.0190 (11)	0.0194 (11)	0.0177 (11)	0.0051 (9)	0.0059 (9)	0.0012 (9)
C4A	0.0165 (10)	0.0194 (11)	0.0105 (10)	0.0080 (9)	0.0020 (8)	−0.0003 (8)
N5	0.0199 (10)	0.0176 (10)	0.0134 (9)	0.0073 (8)	0.0043 (7)	−0.0004 (7)
C5A	0.0139 (10)	0.0181 (11)	0.0145 (10)	0.0063 (8)	−0.0018 (8)	−0.0011 (9)
C6	0.0202 (12)	0.0279 (13)	0.0157 (11)	0.0091 (10)	−0.0001 (9)	−0.0009 (10)
C7	0.0233 (12)	0.0199 (12)	0.0197 (12)	0.0083 (10)	−0.0030 (10)	−0.0068 (9)
C8	0.0205 (12)	0.0200 (12)	0.0245 (13)	0.0051 (9)	−0.0007 (10)	−0.0020 (10)
C9	0.0175 (11)	0.0217 (12)	0.0184 (11)	0.0049 (9)	0.0003 (9)	0.0004 (9)
C9A	0.0151 (10)	0.0167 (11)	0.0143 (10)	0.0067 (8)	−0.0013 (8)	0.0000 (9)
C9B	0.0148 (10)	0.0180 (11)	0.0117 (10)	0.0070 (8)	0.0007 (8)	0.0005 (8)
C10	0.0128 (10)	0.0164 (10)	0.0143 (10)	0.0045 (8)	0.0017 (8)	0.0006 (8)
C10A	0.0129 (10)	0.0164 (10)	0.0136 (10)	0.0046 (8)	0.0014 (8)	−0.0010 (8)
C11	0.0142 (10)	0.0186 (11)	0.0160 (11)	0.0066 (8)	0.0003 (8)	0.0008 (9)
C12	0.0176 (11)	0.0187 (11)	0.0151 (11)	0.0051 (9)	0.0018 (9)	0.0010 (9)
C13	0.0183 (11)	0.0207 (12)	0.0157 (11)	0.0065 (9)	0.0021 (9)	0.0015 (9)
C14	0.0172 (11)	0.0205 (11)	0.0100 (10)	0.0080 (9)	−0.0032 (8)	−0.0034 (8)
C15	0.0192 (11)	0.0171 (11)	0.0172 (11)	0.0049 (9)	0.0004 (9)	0.0003 (9)
C16	0.0191 (11)	0.0200 (11)	0.0140 (10)	0.0072 (9)	0.0009 (8)	0.0007 (9)
C17	0.0206 (11)	0.0237 (12)	0.0148 (11)	0.0049 (9)	0.0080 (9)	0.0024 (9)
C18	0.0275 (13)	0.0243 (12)	0.0186 (12)	0.0020 (10)	0.0115 (10)	−0.0029 (10)
C19	0.0232 (13)	0.0351 (15)	0.0190 (12)	−0.0035 (11)	0.0052 (10)	−0.0055 (11)
C20	0.0191 (12)	0.0409 (15)	0.0143 (11)	0.0041 (11)	0.0046 (9)	0.0027 (11)
C21	0.0208 (12)	0.0319 (14)	0.0152 (11)	0.0070 (10)	0.0039 (9)	0.0039 (10)
C22	0.0201 (11)	0.0215 (12)	0.0143 (10)	0.0031 (9)	0.0029 (9)	0.0012 (9)
C23	0.0214 (13)	0.063 (2)	0.0212 (13)	0.0082 (14)	0.0004 (11)	0.0044 (14)
C24	0.0138 (10)	0.0183 (11)	0.0165 (11)	0.0052 (8)	0.0042 (8)	0.0028 (9)
I2	0.03386 (11)	0.02806 (11)	0.01843 (10)	0.00133 (7)	0.00502 (7)	−0.00585 (7)
S2	0.0219 (3)	0.0174 (3)	0.0221 (3)	0.0065 (2)	0.0062 (2)	0.0012 (2)
O6	0.0238 (9)	0.0233 (9)	0.0166 (8)	0.0103 (7)	−0.0030 (7)	0.0036 (7)
O7	0.0278 (9)	0.0237 (9)	0.0243 (9)	0.0118 (8)	0.0076 (8)	0.0058 (7)
O8	0.0321 (10)	0.0179 (9)	0.0352 (11)	0.0066 (8)	0.0099 (9)	−0.0018 (8)
O9	0.0160 (9)	0.0547 (14)	0.0266 (10)	0.0077 (9)	0.0007 (8)	0.0158 (10)
O10	0.0145 (8)	0.0477 (12)	0.0194 (9)	0.0086 (8)	0.0046 (7)	0.0069 (8)
C25	0.0162 (10)	0.0220 (12)	0.0127 (10)	0.0048 (9)	0.0016 (8)	0.0038 (9)
N26	0.0165 (9)	0.0199 (10)	0.0142 (9)	0.0081 (8)	0.0006 (7)	0.0022 (8)
C27	0.0149 (10)	0.0206 (11)	0.0154 (11)	0.0071 (9)	0.0008 (8)	0.0019 (9)
C27A	0.0140 (10)	0.0196 (11)	0.0129 (10)	0.0055 (8)	0.0011 (8)	0.0018 (8)
C28	0.0178 (11)	0.0201 (11)	0.0177 (11)	0.0073 (9)	0.0003 (9)	0.0003 (9)
C28A	0.0186 (11)	0.0193 (11)	0.0181 (11)	0.0043 (9)	0.0048 (9)	0.0023 (9)
N29	0.0200 (10)	0.0199 (10)	0.0190 (10)	0.0037 (8)	0.0049 (8)	0.0002 (8)
C29A	0.0196 (11)	0.0202 (11)	0.0187 (11)	0.0019 (9)	0.0033 (9)	0.0030 (9)
C30	0.0308 (14)	0.0200 (12)	0.0223 (13)	0.0015 (10)	0.0060 (11)	−0.0011 (10)
C31	0.0363 (16)	0.0246 (13)	0.0188 (12)	−0.0029 (12)	0.0023 (11)	−0.0042 (10)
C32	0.0326 (15)	0.0317 (15)	0.0206 (13)	0.0001 (12)	−0.0021 (11)	−0.0008 (11)
C33	0.0231 (13)	0.0289 (14)	0.0208 (12)	0.0033 (10)	−0.0001 (10)	0.0010 (11)
C33A	0.0190 (11)	0.0213 (12)	0.0169 (11)	0.0019 (9)	0.0031 (9)	0.0001 (9)
C33B	0.0169 (11)	0.0209 (11)	0.0166 (11)	0.0041 (9)	0.0031 (9)	0.0015 (9)
C34	0.0142 (10)	0.0222 (12)	0.0133 (10)	0.0045 (9)	0.0009 (8)	0.0024 (9)
C34A	0.0156 (10)	0.0183 (11)	0.0125 (10)	0.0042 (8)	0.0029 (8)	0.0026 (8)
C35	0.0177 (11)	0.0197 (11)	0.0115 (10)	0.0044 (9)	0.0007 (8)	0.0021 (9)
C36	0.0194 (11)	0.0264 (12)	0.0152 (11)	0.0101 (10)	−0.0014 (9)	0.0012 (10)
C37	0.0273 (13)	0.0261 (13)	0.0181 (12)	0.0137 (10)	0.0023 (10)	0.0020 (10)
C38	0.0252 (12)	0.0232 (12)	0.0105 (10)	0.0043 (10)	0.0009 (9)	−0.0012 (9)
C39	0.0171 (11)	0.0258 (12)	0.0155 (11)	0.0035 (9)	0.0003 (9)	0.0032 (9)
C40	0.0161 (11)	0.0249 (12)	0.0176 (11)	0.0073 (9)	0.0032 (9)	0.0063 (10)
C41	0.0199 (12)	0.0267 (13)	0.0244 (13)	0.0075 (10)	0.0071 (10)	0.0068 (10)
C42	0.0225 (12)	0.0271 (13)	0.0285 (13)	0.0112 (10)	0.0077 (11)	0.0061 (11)
C43	0.0196 (12)	0.0273 (13)	0.0385 (15)	0.0095 (10)	0.0052 (11)	0.0127 (12)
C44	0.0218 (13)	0.0466 (17)	0.0351 (15)	0.0145 (12)	0.0066 (11)	0.0236 (14)
C45	0.0276 (14)	0.060 (2)	0.0230 (14)	0.0096 (14)	0.0073 (11)	0.0097 (14)
C46	0.0285 (14)	0.0398 (16)	0.0253 (14)	0.0066 (12)	0.0070 (11)	0.0012 (12)
C47	0.0283 (15)	0.066 (2)	0.0407 (18)	0.0131 (15)	0.0105 (13)	0.0328 (18)
C48	0.0156 (11)	0.0228 (12)	0.0173 (11)	0.0056 (9)	0.0030 (9)	0.0028 (9)
O11	0.034 (2)	0.048 (3)	0.043 (3)	0.014 (2)	0.008 (2)	0.009 (2)
C49	0.045 (5)	0.055 (5)	0.047 (5)	0.018 (5)	0.023 (4)	0.016 (5)
C50	0.032 (7)	0.044 (4)	0.057 (8)	0.003 (6)	0.022 (4)	0.011 (7)

### (3aSR,10RS,10aRS)-2-(4-Iodophenyl)-1-oxo-5-tosyl-1,2,3,3a,4,5,10,10a-octahydropyrrolo[3,4-b]carbazole-10-carboxylic acid–ethanol (4/1) Geometric parameters (Å, º)

**Table:**

I1—C14	2.094 (2)	S2—C41	1.761 (3)
S1—O2	1.4295 (19)	O6—C25	1.233 (3)
S1—O3	1.4321 (19)	O9—C48	1.205 (3)
S1—N5	1.674 (2)	O10—C48	1.320 (3)
S1—C17	1.755 (3)	O10—H10O	0.78 (5)
O1—C1	1.226 (3)	C25—N26	1.359 (3)
O4—C24	1.211 (3)	C25—C34A	1.505 (3)
O5—C24	1.328 (3)	N26—C35	1.419 (3)
O5—H5O	0.85 (4)	N26—C27	1.485 (3)
C1—N2	1.366 (3)	C27—C27A	1.527 (3)
C1—C10A	1.506 (3)	C27—H27A	0.9900
N2—C11	1.415 (3)	C27—H27B	0.9900
N2—C3	1.489 (3)	C27A—C28	1.525 (3)
C3—C3A	1.534 (3)	C27A—C34A	1.534 (3)
C3—H3A	0.9900	C27A—H27C	1.0000
C3—H3B	0.9900	C28—C28A	1.505 (3)
C3A—C10A	1.523 (3)	C28—H28A	0.9900
C3A—C4	1.528 (3)	C28—H28B	0.9900
C3A—H3C	1.0000	C28A—C33B	1.364 (3)
C4—C4A	1.505 (3)	C28A—N29	1.423 (3)
C4—H4A	0.9900	N29—C29A	1.421 (3)
C4—H4B	0.9900	C29A—C30	1.394 (4)
C4A—C9B	1.363 (3)	C29A—C33A	1.410 (4)
C4A—N5	1.426 (3)	C30—C31	1.388 (4)
N5—C5A	1.424 (3)	C30—H30	0.9500
C5A—C6	1.395 (3)	C31—C32	1.400 (5)
C5A—C9A	1.404 (3)	C31—H31	0.9500
C6—C7	1.398 (4)	C32—C33	1.383 (4)
C6—H6	0.9500	C32—H32	0.9500
C7—C8	1.404 (4)	C33—C33A	1.400 (4)
C7—H7	0.9500	C33—H33	0.9500
C8—C9	1.385 (4)	C33A—C33B	1.441 (3)
C8—H8	0.9500	C33B—C34	1.512 (3)
C9—C9A	1.396 (4)	C34—C34A	1.524 (3)
C9—H9	0.9500	C34—C48	1.529 (3)
C9A—C9B	1.448 (3)	C34—H34	1.0000
C9B—C10	1.514 (3)	C34A—H34A	1.0000
C10—C10A	1.523 (3)	C35—C40	1.396 (3)
C10—C24	1.530 (3)	C35—C36	1.399 (3)
C10—H10	1.0000	C36—C37	1.392 (4)
C10A—H10A	1.0000	C36—H36	0.9500
C11—C12	1.388 (3)	C37—C38	1.389 (4)
C11—C16	1.403 (3)	C37—H37	0.9500
C12—C13	1.402 (3)	C38—C39	1.392 (4)
C12—H12	0.9500	C39—C40	1.386 (4)
C13—C14	1.387 (4)	C39—H39	0.9500
C13—H13	0.9500	C40—H40	0.9500
C14—C15	1.386 (4)	C41—C42	1.382 (4)
C15—C16	1.399 (3)	C41—C46	1.398 (4)
C15—H15	0.9500	C42—C43	1.391 (4)
C16—H16	0.9500	C42—H42	0.9500
C17—C22	1.388 (4)	C43—C44	1.393 (5)
C17—C18	1.393 (4)	C43—H43	0.9500
C18—C19	1.388 (4)	C44—C45	1.394 (5)
C18—H18	0.9500	C44—C47	1.514 (4)
C19—C20	1.401 (4)	C45—C46	1.391 (5)
C19—H19	0.9500	C45—H45	0.9500
C20—C21	1.396 (4)	C46—H46	0.9500
C20—C23	1.504 (4)	C47—H47A	0.9800
C21—C22	1.383 (4)	C47—H47B	0.9800
C21—H21	0.9500	C47—H47C	0.9800
C22—H22	0.9500	O11—C49	1.428 (3)
C23—H23A	0.9800	O11—H11O	0.9000
C23—H23B	0.9800	C49—C50	1.523 (3)
C23—H23C	0.9800	C49—H49A	0.9900
I2—C38	2.091 (3)	C49—H49B	0.9900
S2—O7	1.424 (2)	C50—H50A	0.9800
S2—O8	1.433 (2)	C50—H50B	0.9800
S2—N29	1.673 (2)	C50—H50C	0.9800
			
O2—S1—O3	120.60 (11)	O6—C25—N26	125.5 (2)
O2—S1—N5	106.39 (11)	O6—C25—C34A	126.9 (2)
O3—S1—N5	105.96 (12)	N26—C25—C34A	107.6 (2)
O2—S1—C17	109.32 (13)	C25—N26—C35	126.0 (2)
O3—S1—C17	108.72 (12)	C25—N26—C27	111.6 (2)
N5—S1—C17	104.67 (11)	C35—N26—C27	121.86 (19)
C24—O5—H5O	105 (3)	N26—C27—C27A	101.99 (18)
O1—C1—N2	127.8 (2)	N26—C27—H27A	111.4
O1—C1—C10A	124.3 (2)	C27A—C27—H27A	111.4
N2—C1—C10A	107.8 (2)	N26—C27—H27B	111.4
C1—N2—C11	124.9 (2)	C27A—C27—H27B	111.4
C1—N2—C3	111.85 (19)	H27A—C27—H27B	109.2
C11—N2—C3	123.3 (2)	C28—C27A—C27	116.95 (19)
N2—C3—C3A	101.80 (19)	C28—C27A—C34A	111.18 (19)
N2—C3—H3A	111.4	C27—C27A—C34A	100.81 (19)
C3A—C3—H3A	111.4	C28—C27A—H27C	109.2
N2—C3—H3B	111.4	C27—C27A—H27C	109.2
C3A—C3—H3B	111.4	C34A—C27A—H27C	109.2
H3A—C3—H3B	109.3	C28A—C28—C27A	107.44 (19)
C10A—C3A—C4	110.36 (19)	C28A—C28—H28A	110.2
C10A—C3A—C3	102.44 (19)	C27A—C28—H28A	110.2
C4—C3A—C3	119.7 (2)	C28A—C28—H28B	110.2
C10A—C3A—H3C	107.9	C27A—C28—H28B	110.2
C4—C3A—H3C	107.9	H28A—C28—H28B	108.5
C3—C3A—H3C	107.9	C33B—C28A—N29	108.7 (2)
C4A—C4—C3A	107.1 (2)	C33B—C28A—C28	126.7 (2)
C4A—C4—H4A	110.3	N29—C28A—C28	124.6 (2)
C3A—C4—H4A	110.3	C29A—N29—C28A	107.8 (2)
C4A—C4—H4B	110.3	C29A—N29—S2	123.48 (18)
C3A—C4—H4B	110.3	C28A—N29—S2	124.75 (18)
H4A—C4—H4B	108.5	C30—C29A—C33A	121.3 (2)
C9B—C4A—N5	108.5 (2)	C30—C29A—N29	131.3 (2)
C9B—C4A—C4	126.9 (2)	C33A—C29A—N29	107.4 (2)
N5—C4A—C4	124.3 (2)	C31—C30—C29A	117.3 (3)
C5A—N5—C4A	107.96 (19)	C31—C30—H30	121.4
C5A—N5—S1	123.83 (17)	C29A—C30—H30	121.4
C4A—N5—S1	123.68 (17)	C30—C31—C32	121.9 (3)
C6—C5A—C9A	121.9 (2)	C30—C31—H31	119.0
C6—C5A—N5	130.5 (2)	C32—C31—H31	119.0
C9A—C5A—N5	107.5 (2)	C33—C32—C31	120.8 (3)
C5A—C6—C7	116.6 (2)	C33—C32—H32	119.6
C5A—C6—H6	121.7	C31—C32—H32	119.6
C7—C6—H6	121.7	C32—C33—C33A	118.3 (3)
C6—C7—C8	122.0 (2)	C32—C33—H33	120.9
C6—C7—H7	119.0	C33A—C33—H33	120.9
C8—C7—H7	119.0	C33—C33A—C29A	120.4 (2)
C9—C8—C7	120.5 (3)	C33—C33A—C33B	132.2 (3)
C9—C8—H8	119.8	C29A—C33A—C33B	107.3 (2)
C7—C8—H8	119.8	C28A—C33B—C33A	108.6 (2)
C8—C9—C9A	118.6 (2)	C28A—C33B—C34	124.7 (2)
C8—C9—H9	120.7	C33A—C33B—C34	126.4 (2)
C9A—C9—H9	120.7	C33B—C34—C34A	105.51 (19)
C9—C9A—C5A	120.4 (2)	C33B—C34—C48	112.4 (2)
C9—C9A—C9B	132.3 (2)	C34A—C34—C48	115.9 (2)
C5A—C9A—C9B	107.3 (2)	C33B—C34—H34	107.5
C4A—C9B—C9A	108.8 (2)	C34A—C34—H34	107.5
C4A—C9B—C10	123.1 (2)	C48—C34—H34	107.5
C9A—C9B—C10	127.6 (2)	C25—C34A—C34	119.7 (2)
C9B—C10—C10A	105.48 (19)	C25—C34A—C27A	102.91 (18)
C9B—C10—C24	112.02 (18)	C34—C34A—C27A	114.4 (2)
C10A—C10—C24	110.88 (19)	C25—C34A—H34A	106.3
C9B—C10—H10	109.5	C34—C34A—H34A	106.3
C10A—C10—H10	109.5	C27A—C34A—H34A	106.3
C24—C10—H10	109.5	C40—C35—C36	119.8 (2)
C1—C10A—C3A	104.07 (19)	C40—C35—N26	118.8 (2)
C1—C10A—C10	118.0 (2)	C36—C35—N26	121.4 (2)
C3A—C10A—C10	111.74 (19)	C37—C36—C35	119.7 (2)
C1—C10A—H10A	107.5	C37—C36—H36	120.1
C3A—C10A—H10A	107.5	C35—C36—H36	120.1
C10—C10A—H10A	107.5	C38—C37—C36	120.0 (2)
C12—C11—C16	119.4 (2)	C38—C37—H37	120.0
C12—C11—N2	120.5 (2)	C36—C37—H37	120.0
C16—C11—N2	120.1 (2)	C37—C38—C39	120.4 (2)
C11—C12—C13	120.7 (2)	C37—C38—I2	120.04 (19)
C11—C12—H12	119.6	C39—C38—I2	119.56 (19)
C13—C12—H12	119.6	C40—C39—C38	119.8 (2)
C14—C13—C12	119.4 (2)	C40—C39—H39	120.1
C14—C13—H13	120.3	C38—C39—H39	120.1
C12—C13—H13	120.3	C39—C40—C35	120.3 (2)
C15—C14—C13	120.5 (2)	C39—C40—H40	119.9
C15—C14—I1	121.75 (18)	C35—C40—H40	119.9
C13—C14—I1	117.60 (18)	C42—C41—C46	121.3 (3)
C14—C15—C16	120.2 (2)	C42—C41—S2	119.1 (2)
C14—C15—H15	119.9	C46—C41—S2	119.6 (2)
C16—C15—H15	119.9	C41—C42—C43	118.9 (3)
C15—C16—C11	119.7 (2)	C41—C42—H42	120.6
C15—C16—H16	120.1	C43—C42—H42	120.6
C11—C16—H16	120.1	C42—C43—C44	121.2 (3)
C22—C17—C18	121.3 (2)	C42—C43—H43	119.4
C22—C17—S1	118.7 (2)	C44—C43—H43	119.4
C18—C17—S1	119.9 (2)	C43—C44—C45	118.8 (3)
C19—C18—C17	118.5 (3)	C43—C44—C47	119.7 (3)
C19—C18—H18	120.8	C45—C44—C47	121.5 (3)
C17—C18—H18	120.8	C46—C45—C44	121.0 (3)
C18—C19—C20	121.4 (3)	C46—C45—H45	119.5
C18—C19—H19	119.3	C44—C45—H45	119.5
C20—C19—H19	119.3	C45—C46—C41	118.8 (3)
C21—C20—C19	118.4 (3)	C45—C46—H46	120.6
C21—C20—C23	120.4 (3)	C41—C46—H46	120.6
C19—C20—C23	121.2 (3)	C44—C47—H47A	109.5
C22—C21—C20	121.1 (3)	C44—C47—H47B	109.5
C22—C21—H21	119.5	H47A—C47—H47B	109.5
C20—C21—H21	119.5	C44—C47—H47C	109.5
C21—C22—C17	119.3 (2)	H47A—C47—H47C	109.5
C21—C22—H22	120.4	H47B—C47—H47C	109.5
C17—C22—H22	120.4	O9—C48—O10	123.9 (2)
C20—C23—H23A	109.5	O9—C48—C34	122.7 (2)
C20—C23—H23B	109.5	O10—C48—C34	113.5 (2)
H23A—C23—H23B	109.5	C49—O11—H11O	109.7
C20—C23—H23C	109.5	O11—C49—C50	112.5 (3)
H23A—C23—H23C	109.5	O11—C49—H49A	109.1
H23B—C23—H23C	109.5	C50—C49—H49A	109.1
O4—C24—O5	123.9 (2)	O11—C49—H49B	109.1
O4—C24—C10	124.1 (2)	C50—C49—H49B	109.1
O5—C24—C10	112.0 (2)	H49A—C49—H49B	107.8
O7—S2—O8	120.30 (13)	C49—C50—H50A	109.5
O7—S2—N29	106.29 (11)	C49—C50—H50B	109.5
O8—S2—N29	105.63 (12)	H50A—C50—H50B	109.5
O7—S2—C41	108.47 (13)	C49—C50—H50C	109.5
O8—S2—C41	109.40 (13)	H50A—C50—H50C	109.5
N29—S2—C41	105.79 (12)	H50B—C50—H50C	109.5
C48—O10—H10O	109 (3)		
			
O1—C1—N2—C11	5.3 (4)	O6—C25—N26—C35	−5.6 (4)
C10A—C1—N2—C11	−178.8 (2)	C34A—C25—N26—C35	170.8 (2)
O1—C1—N2—C3	−173.0 (2)	O6—C25—N26—C27	−177.1 (2)
C10A—C1—N2—C3	2.9 (3)	C34A—C25—N26—C27	−0.7 (3)
C1—N2—C3—C3A	−23.2 (2)	C25—N26—C27—C27A	−23.1 (3)
C11—N2—C3—C3A	158.5 (2)	C35—N26—C27—C27A	165.0 (2)
N2—C3—C3A—C10A	33.0 (2)	N26—C27—C27A—C28	156.6 (2)
N2—C3—C3A—C4	155.4 (2)	N26—C27—C27A—C34A	36.0 (2)
C10A—C3A—C4—C4A	−43.7 (2)	C27—C27A—C28—C28A	−157.2 (2)
C3—C3A—C4—C4A	−162.1 (2)	C34A—C27A—C28—C28A	−42.2 (3)
C3A—C4—C4A—C9B	10.2 (3)	C27A—C28—C28A—C33B	9.4 (4)
C3A—C4—C4A—N5	−177.3 (2)	C27A—C28—C28A—N29	−169.4 (2)
C9B—C4A—N5—C5A	−1.6 (3)	C33B—C28A—N29—C29A	2.7 (3)
C4—C4A—N5—C5A	−175.2 (2)	C28—C28A—N29—C29A	−178.4 (2)
C9B—C4A—N5—S1	−158.37 (17)	C33B—C28A—N29—S2	160.67 (19)
C4—C4A—N5—S1	27.9 (3)	C28—C28A—N29—S2	−20.4 (4)
O2—S1—N5—C5A	164.11 (19)	O7—S2—N29—C29A	−170.7 (2)
O3—S1—N5—C5A	34.6 (2)	O8—S2—N29—C29A	−41.9 (2)
C17—S1—N5—C5A	−80.2 (2)	C41—S2—N29—C29A	74.1 (2)
O2—S1—N5—C4A	−42.7 (2)	O7—S2—N29—C28A	34.6 (2)
O3—S1—N5—C4A	−172.16 (18)	O8—S2—N29—C28A	163.4 (2)
C17—S1—N5—C4A	73.0 (2)	C41—S2—N29—C28A	−80.6 (2)
C4A—N5—C5A—C6	177.9 (2)	C28A—N29—C29A—C30	176.8 (3)
S1—N5—C5A—C6	−25.3 (4)	S2—N29—C29A—C30	18.4 (4)
C4A—N5—C5A—C9A	1.6 (2)	C28A—N29—C29A—C33A	−3.6 (3)
S1—N5—C5A—C9A	158.34 (17)	S2—N29—C29A—C33A	−161.97 (18)
C9A—C5A—C6—C7	0.4 (4)	C33A—C29A—C30—C31	−2.5 (4)
N5—C5A—C6—C7	−175.5 (2)	N29—C29A—C30—C31	177.0 (3)
C5A—C6—C7—C8	−0.2 (4)	C29A—C30—C31—C32	2.0 (4)
C6—C7—C8—C9	−0.2 (4)	C30—C31—C32—C33	−0.6 (5)
C7—C8—C9—C9A	0.3 (4)	C31—C32—C33—C33A	−0.2 (5)
C8—C9—C9A—C5A	0.0 (3)	C32—C33—C33A—C29A	−0.4 (4)
C8—C9—C9A—C9B	176.6 (2)	C32—C33—C33A—C33B	178.3 (3)
C6—C5A—C9A—C9	−0.3 (3)	C30—C29A—C33A—C33	1.8 (4)
N5—C5A—C9A—C9	176.4 (2)	N29—C29A—C33A—C33	−177.8 (2)
C6—C5A—C9A—C9B	−177.7 (2)	C30—C29A—C33A—C33B	−177.2 (2)
N5—C5A—C9A—C9B	−1.0 (2)	N29—C29A—C33A—C33B	3.2 (3)
N5—C4A—C9B—C9A	0.9 (3)	N29—C28A—C33B—C33A	−0.7 (3)
C4—C4A—C9B—C9A	174.4 (2)	C28—C28A—C33B—C33A	−179.6 (2)
N5—C4A—C9B—C10	−171.2 (2)	N29—C28A—C33B—C34	−175.0 (2)
C4—C4A—C9B—C10	2.3 (4)	C28—C28A—C33B—C34	6.1 (4)
C9—C9A—C9B—C4A	−176.9 (2)	C33—C33A—C33B—C28A	179.6 (3)
C5A—C9A—C9B—C4A	0.0 (3)	C29A—C33A—C33B—C28A	−1.6 (3)
C9—C9A—C9B—C10	−5.2 (4)	C33—C33A—C33B—C34	−6.2 (5)
C5A—C9A—C9B—C10	171.7 (2)	C29A—C33A—C33B—C34	172.6 (2)
C4A—C9B—C10—C10A	18.9 (3)	C28A—C33B—C34—C34A	12.7 (3)
C9A—C9B—C10—C10A	−151.7 (2)	C33A—C33B—C34—C34A	−160.7 (2)
C4A—C9B—C10—C24	−101.9 (3)	C28A—C33B—C34—C48	−114.6 (3)
C9A—C9B—C10—C24	87.5 (3)	C33A—C33B—C34—C48	72.1 (3)
O1—C1—C10A—C3A	−165.0 (2)	O6—C25—C34A—C34	−31.2 (4)
N2—C1—C10A—C3A	18.9 (2)	N26—C25—C34A—C34	152.5 (2)
O1—C1—C10A—C10	−40.5 (3)	O6—C25—C34A—C27A	−159.5 (2)
N2—C1—C10A—C10	143.4 (2)	N26—C25—C34A—C27A	24.2 (2)
C4—C3A—C10A—C1	−160.65 (19)	C33B—C34—C34A—C25	−170.2 (2)
C3—C3A—C10A—C1	−32.1 (2)	C48—C34—C34A—C25	−45.1 (3)
C4—C3A—C10A—C10	71.0 (2)	C33B—C34—C34A—C27A	−47.4 (3)
C3—C3A—C10A—C10	−160.51 (19)	C48—C34—C34A—C27A	77.7 (3)
C9B—C10—C10A—C1	−174.16 (19)	C28—C27A—C34A—C25	−161.5 (2)
C24—C10—C10A—C1	−52.7 (3)	C27—C27A—C34A—C25	−36.9 (2)
C9B—C10—C10A—C3A	−53.6 (2)	C28—C27A—C34A—C34	67.0 (3)
C24—C10—C10A—C3A	67.9 (2)	C27—C27A—C34A—C34	−168.4 (2)
C1—N2—C11—C12	20.6 (3)	C25—N26—C35—C40	−151.0 (2)
C3—N2—C11—C12	−161.3 (2)	C27—N26—C35—C40	19.7 (3)
C1—N2—C11—C16	−161.4 (2)	C25—N26—C35—C36	31.4 (4)
C3—N2—C11—C16	16.8 (3)	C27—N26—C35—C36	−157.9 (2)
C16—C11—C12—C13	−0.9 (4)	C40—C35—C36—C37	−1.2 (4)
N2—C11—C12—C13	177.2 (2)	N26—C35—C36—C37	176.3 (2)
C11—C12—C13—C14	−0.7 (4)	C35—C36—C37—C38	0.3 (4)
C12—C13—C14—C15	1.3 (4)	C36—C37—C38—C39	0.8 (4)
C12—C13—C14—I1	−174.40 (17)	C36—C37—C38—I2	−176.9 (2)
C13—C14—C15—C16	−0.4 (4)	C37—C38—C39—C40	−1.0 (4)
I1—C14—C15—C16	175.14 (18)	I2—C38—C39—C40	176.72 (19)
C14—C15—C16—C11	−1.2 (4)	C38—C39—C40—C35	0.1 (4)
C12—C11—C16—C15	1.8 (3)	C36—C35—C40—C39	1.0 (4)
N2—C11—C16—C15	−176.3 (2)	N26—C35—C40—C39	−176.6 (2)
O2—S1—C17—C22	−169.95 (19)	O7—S2—C41—C42	−31.2 (2)
O3—S1—C17—C22	−36.5 (2)	O8—S2—C41—C42	−164.2 (2)
N5—S1—C17—C22	76.4 (2)	N29—S2—C41—C42	82.5 (2)
O2—S1—C17—C18	9.5 (2)	O7—S2—C41—C46	146.3 (2)
O3—S1—C17—C18	143.0 (2)	O8—S2—C41—C46	13.3 (3)
N5—S1—C17—C18	−104.1 (2)	N29—S2—C41—C46	−100.0 (2)
C22—C17—C18—C19	0.6 (4)	C46—C41—C42—C43	−0.4 (4)
S1—C17—C18—C19	−178.9 (2)	S2—C41—C42—C43	177.1 (2)
C17—C18—C19—C20	0.9 (4)	C41—C42—C43—C44	−1.8 (4)
C18—C19—C20—C21	−1.6 (4)	C42—C43—C44—C45	3.1 (4)
C18—C19—C20—C23	179.5 (3)	C42—C43—C44—C47	−175.2 (3)
C19—C20—C21—C22	0.8 (4)	C43—C44—C45—C46	−2.1 (5)
C23—C20—C21—C22	179.8 (3)	C47—C44—C45—C46	176.1 (3)
C20—C21—C22—C17	0.6 (4)	C44—C45—C46—C41	0.0 (5)
C18—C17—C22—C21	−1.3 (4)	C42—C41—C46—C45	1.3 (4)
S1—C17—C22—C21	178.16 (19)	S2—C41—C46—C45	−176.1 (2)
C9B—C10—C24—O4	124.7 (3)	C33B—C34—C48—O9	−92.4 (3)
C10A—C10—C24—O4	7.2 (3)	C34A—C34—C48—O9	146.2 (3)
C9B—C10—C24—O5	−54.7 (3)	C33B—C34—C48—O10	86.9 (3)
C10A—C10—C24—O5	−172.29 (19)	C34A—C34—C48—O10	−34.6 (3)

### (3aSR,10RS,10aRS)-2-(4-Iodophenyl)-1-oxo-5-tosyl-1,2,3,3a,4,5,10,10a-octahydropyrrolo[3,4-b]carbazole-10-carboxylic acid–ethanol (4/1) Hydrogen-bond geometry (Å, º)

*Cg*6, *Cg*8 and *Cg*12 are the centroids of the C5*A*/C6–C9/C9*A*, C17–C22 and C41–C46 rings, respectively.

**Table:**

*D*—H···*A*	*D*—H	H···*A*	*D*···*A*	*D*—H···*A*
O5—H5*O*···O6	0.85 (4)	1.80 (4)	2.640 (2)	174 (4)
C3*A*—H3*C*···O4	1.00	2.44	3.066 (3)	120
C6—H6···O3	0.95	2.32	2.913 (4)	120
C12—H12···O1	0.95	2.23	2.838 (3)	121
C18—H18···O8^i^	0.95	2.45	3.298 (3)	148
O10—H10*O*···O1^ii^	0.78 (5)	1.87 (5)	2.623 (3)	163 (5)
C27*A*—H27*C*···O10	1.00	2.35	3.011 (3)	123
C30—H30···O8	0.95	2.40	2.980 (4)	119
C36—H36···O6	0.95	2.35	2.895 (3)	116
O11—H11*O*···O5^ii^	0.90	2.12	3.024 (6)	177
C27—H27*A*···*Cg*8^iii^	0.99	2.87	3.729 (3)	145
C37—H37···*Cg*12^iv^	0.95	2.93	3.776 (3)	148
C39—H39···*Cg*6^iii^	0.95	2.70	3.526 (3)	145

Symmetry codes: (i) *x*, *y*+1, *z*; (ii) −*x*+1, −*y*+1, −*z*+1; (iii) *x*+1, *y*, *z*; (iv) −*x*+2, −*y*+1, −*z*+1.
