# Supplementary material for: Synthesis, crystal structure and Hirshfeld surface analysis of (3aSR,10RS,10aRS)-2-(4-iodo­phen­yl)-1-oxo-5-tosyl-1,2,3,3a,4,5,10,10a-octa­hydro­pyrrolo[3,4-b]carbazole-10-carb­oxy­lic acid–ethanol (4/1)
Source: Acta Crystallogr E Crystallogr Commun. 2026 Jan 6;82(Pt 2):126–31. doi: 10.1107/S2056989025011582 (PMC12874240; doi:10.1107/S2056989025011582)
Supplement: Supplementary file 3 [file e-82-00126-sup3.pdf]

Table S1. Selected interatomic distances (Å).

S1···H4B	3.04	H4B···O8^{iv}	2.68
O1···C12	2.838 (3)	H18···O8^{iv}	2.46
O2···C4	2.868 (3)	O9···H12^{i}	2.62
O3···C6	2.913 (4)	O10···H27C	2.35
O4···C3A	3.066 (3)	O10···H23C^{iii}	2.68
O4···C1	2.798 (3)	O11···H10^{i}	2.71
O5···O11^{i}	3.024 (6)	O11···H43^{iii}	2.60
O6···C36	2.894 (3)	C12···C43^{iii}	3.216 (4)
O7···C28	2.817 (3)	C12···C42^{iii}	3.238 (4)
O8···C30	2.980 (4)	C21···C40^{iii}	3.387 (4)
O10···C27A	3.011 (3)	C21···C35^{iii}	3.304 (4)
O10···C25	3.100 (4)	C1···H12	2.64
O1···H12	2.23	C3···H16	2.64
O1···H10O^{i}	1.87 (5)	C4A···H10A	2.76
O1···H49A^{i}	2.65	C7···H39^{iii}	2.87
O1···H50B	2.60	C8···H39^{iii}	2.80
O2···H18	2.57	C10···H11O^{i}	2.83
O2···H4B	2.51	C16···H3A	2.82
H30···O2^{ii}	2.64	C18···H27A^{iii}	2.89
O3···H6	2.32	C19···H27A^{iii}	2.83
O3···H22	2.70	C24···H27B^{iii}	2.73
O4···H50C^{i}	2.63	C24···H50C^{i}	2.71
O4···H42^{iii}	2.68	C24···H11O^{i}	2.51
O4···H27B^{iii}	2.71	C24···H3C	2.64
O4···H3C	2.44	C25···H36	2.77
O4···H49B	2.57	C27···H40	2.57
O5···H27B^{iii}	2.71	C28A···H34A	2.80
O5···H40^{iii}	2.64	C35···H34A^{iii}	2.81
O5···H11O^{i}	2.12	C40···H27A	2.84
O6···H5O	1.80 (4)	C40···H34^{iii}	2.81
O6···H36	2.35	C43···H37^{iii}	2.73
O7···H28A	2.71	H10···H11O^{i}	2.31
O7···H3A^{iii}	2.51	H11O···H50C	2.27
O7···H42	2.66	H16···H3A	2.40
O7···H28B	2.44	H21···H23B	2.35
O8···H46	2.59	H27B···H40	2.40
O8···H30	2.40	H45···H47C	2.38

Symmetry codes: (i) $-x+1, -y+1, -z+1$; (ii) $x, y-1, z$; (iii) $-x+2, -y+1, -z+1$; (iv) $x, y+1, z$.

Table S2. Comparison of the percentages for molecules a and b.

Contacts	**a**	**b**
H···H	36.1	38.5
H···O/O···H	23.7	22.1
H···C/C···H	20.0	16.1
H···I/I···H	6.4	10.1
C···I/I···C	5.0	2.0
C···C	4.0	5.8
H···N/N···H	1.5	1.7
I···I	1.1	1.1
O···0	0.8	0.2
C···O/O···C	0.7	0.7
O···I/I···O	0.5	0.0
N···O/O···N	0.2	0.0
C···N/N···C	0.1	1.4
